# Household Hints and Recipes

**Published:** 1876-01

**Authors:** 


### Household Hints & Recipes.

TO PRESERVE FENCE POSTS.—According to excellent authority, fence posts can be made as imperishable as iron for less than two cents apiece. Pulverized charcoal is to be stirred into boiled linseed oil to the consistency of paint, and a coat of this to be brushed over the part of the post to be placed in the ground.

POCKET MUCILAGE.—Boil one pound of the best white glue and strain very clear; boil also four ounces of isinglass, and mix the two together: place them on a water-bath with half a pound of white sugar, and evaporate until the liquid is quite thick, when it is to be poured into moulds, cut, and dried to carry in the pocket. This mucilage immediately dissolves in water, and fastens paper very firmly.

CEMENT FOR IRON.—Take equal parts of sulphur and white lead, with about a sixth of borax; incorporate them so as to form one homogeneous mass. When going to apply, wet it with strong sulphuric acid, and place a thin layer of it between the two pieces of iron, which should then be pressed together. In five days it will be perfectly dry, all traces of the cement having vanished, and the iron will have the appearance of having been welded together.

MARKING TOOLS.—Much trouble can often be saved by marking tools with the owners' names, which can easily be done in the following manner: Coat the tools with a thin layer of wax or hard tallow, by first warming the steel and rubbing on the wax warm until it flows, and let it cool. When hard, mark the name through the wax with a graver, and apply nitric acid; after a few moments wash off the acid, and wipe off with a soft rag, when the letters will be etched into the steel.

ELECTROPLATING PEWTER SURFACES.—Take one ounce nitric acid and drop pieces of copper in it until effervescence ceases; then add one-half ounce water, and the solution is ready for use. Place a few drops of the solution on the desired surface, and touch it with a piece of steel, and there will be a beautiful film of copper deposited. The application may be repeated if necessary, though once is generally sufficient. The article must now be washed, and immediately placed in the plating bath, when deposition will take place with perfect ease.—*Scientific American.*

TO DYE IVORY BLUE.—By keeping the ivory immersed in a dilute solution of sulphate of indigo, partly saturated with potash, for some time, a fine blue color will be given to it.

IMPROVED STARCH.—A beautiful finish can be given to articles to be starched by taking one fourth of a pound of starch, and working it over and kneading it with a little water, then placing five or six pints of water in a pan, and adding to this a very small quantity of powdered borax, a small piece of sugar, and a fragment of white wax about the size of a hazel nut, and heating the whole sufficiently. This water is then to be added to the starch, with continued stirring, mixing the two together until the whole is as thick as is convenient for application. If the articles are to be made quite stiff, the strength of the starch may be increased two or three fold.

TO PRESERVE A BOUQUET.—The *American Artisan* says; "When you receive a bouquet, sprinkle it with fresh water; then put it into a vessel containing some soap-suds, which nourish the roots and keep the flowers as good as new. Take the bouquet out of the suds every morning, and lay it sideways in fresh water, the stock entering first into the water. Keep it there a minute or two, then take it out and sprinkle the flowers lightly by the hand with pure water. Replace the bouquet in soap-suds, and the flowers will bloom as fresh as when gathered. The soap-suds need to be changed every third day. By observing these rules, a bouquet may be kept bright and beautiful for at least one month, and will last longer in a very passable state; but the attention to the fair but frail creatures, as directed above, must be strictly observed, or 'the last rose of summer' will not be 'left blooming alone,' but will perish."

MILK KEPT BY CHLOROFORM.—That milk can be kept sweet by the addition of a little chloroform is a suggestion for which we have to thank J. P. Barnes, of London. When added in sufficient quantity to fresh milk, the lactic fermentation is prevented. To eight fluid ounces of fresh milk was added respectively, ten and twenty minims of chloroform; they were kept in a warm place and occasionally agitated; after five days had elapsed, that containing ten minums had developed lactic acid in quantity sufficient to separate the casein, while that containing twenty remained fresh and good. It might be found convenient to preserve milk in this manner, always taking care to boil it just before using, in order to drive off the chloroform.

EFFECT OF AMMONIA FUMES ON FLOWERS.—It is well known that the smoke of tobacco will, when applied in sufficient quantity, change the tint of flowers; but Professor Gabba experiments by pouring a little ammonia liquor into a saucer and inverting a funnel over it. Placing the flowers in the tube of the latter, he finds that blue, violet, and purple-colored blossoms become of a fine green; carmine and crimson become black; white, yellow; while particolored flowers, such as red and white, are changed to green and yellow. If the flowers are immersed in water, the natural color will return in a few hours. Professor Gabba also found that asters acquire a pleasing odor when submitted to the fumes of ammonia.

---

HOW TO EXTINGUISH LAMPS WITH CHIMNEYS.—A correspondent of the *English Mechanic*, says: "Turn the flame up to full power, then blow a sharp puff *horizontally* across the top of the funnel, when the light will not only be extinguished, but there will be no after-smoke—the formerly ignited wick will be extinguished by its own carbonic acid gas. On leaving my office at night I thus turn up the flaming wick, and, with a grateful gladness that the desk labors of the day (and night) are over, give a side wave of the hat past the chimney, which draws up the flame from contact with the wick, and the light is gone, and with no after-smell. This cannot be too widely circulated, as I read in the *Times* the other day, that a lady lost her life by blowing *down* the chimney, and thus causing an explosion.

---

MAKING COFFEE.—We learn from the *Causeries Scientifiques* that M. Doyen has been investigating the subject, and proposed the following method: He uses 15 grammes (or about half an ounce avoirdupois) for two cups. The berries are to be powdered just before they are used. Three fourths of the powder is thrown into cold water, which is made to boil, and kept boiling for ten minutes. Then the remaining fourth of the powder is cast in; the pot is removed from the fire, covered up, and allowed to remain five minutes. The liquid is now ready; but it may, if desired, be passed through linen. So prepared it is brownish,, not black, and slightly turbid from the fatty matter, of which coffee contains 12 per cent. When coffee has to be carried on a journey, as by an army on the march, M. Doyen has the roasted berries ground into an impalpable powder, which is then slightly moistened, combined with twice its weight of sugar, and pressed into tablets like chocolate. These are dried and wrapped in tinfoil, and the coffee ration thus prepared may be used very speedily; for if cast into boiling water the coffee is ready. Precious time and the necessity of having coffee-mills are thus saved.

---

DELICACIES FOR THE SICK.—The following practical receipts from the *Druggists' Circular*, will doubtless prove of use to many of our readers:

CHICKEN JELLY.

Half a raw chicken, pounded with a mallet, bones and meat together:

Cold water to cover it well.

Heat slowly in a covered vessel, and let it simmer until the meat is in white rags and the liquid reduced one-half; strain and press through a coarse cloth; season to taste, return to fire, and simmer five minutes longer; skim when cool.—Give to patient cold, with unleavened wafers.

ARROWROOT JELLY.

One cup of boiling water;
Two teaspoonfuls of Bermuda arrowroot;
One teaspoonful of lemon juice;
Two teaspoonfuls of white sugar.

Wet the arrowroot in a little cold water, and rub smooth; then stir into the hot water, which should be on the fire and actually boiling at the time, with the sugar already melted in it. Stir until clear, boiling steadily all the time, and add the lemon. Wet a cup in cold water, and pour in the jelly to form. Eat cold with sugar and cream.

ARROWROOT WINE JELLY.

One cup of boiling water;
Two teaspoonfuls of arrowroot;
Two teaspoonfuls of white sugar;
One teaspoonful of brandy or three of wine.

Proceed as before. An excellent corrective for weak bowels.

FLAX-SEED LEMONADE.

Four tablespoonfuls of flax-seed, (whole);
One quart of boiling water;
Juice of two lemons.

Pour the boiling water upon the flax-seed, and steep three hours in a covered vessel; sweeten to taste; if too thick, add cold water with the lemon juice. Ice for drinking. It is admirable for colds.

ICELAND MOSS JELLY.

One handful of moss, well washed;
One quart of boiling water;
The juice of two lemons;
One glass of wine;
One quarter of a teaspoonful of cinnamon.

Stir the moss (after soaking it an hour in a little cold water), into the boiling water, and simmer until it is dissolved; sweeten, flavor and strain into moulds. Good for colds and very nourishing.

CHAPPED HANDS.—A little pot of strained honey is to be kept near the wash-stand, and the sufferer, after washing his hands in the usual manner, but before they are dry, is to take a little honey on the end of his finger and to anoint both hands thoroughly. If a proper quantity be used, the hands will not become sticky; they should be well dried before exposure to the winds.

---

LIQUID HARNESS POLISH.—Dissolve shellac in strong alcohol, add a small quantity of camphor, and enough of lamp-black to color it. The proportions may vary according to the kind of harness treated, but are easily ascertained by a little experimenting. The liquid may be applied with a piece of flannel or a soft brush, and requires no rubbing or brushing to bring out the polish. But, in fact, we think this and all similar varnish bad for leather.

---

HAIR RESTORER.—

Bay Rum, 2 oz.;
Glycerine, 2 oz.;
Tincture of cantharides, 1 dr.;
Oil of bergamot, 30 drops;
Sulphate of quinia, 10 grains;
Water, 4 oz. Mix.

Use every morning for dressing the hair, in place of hair-oil or pomatum.

---

BLACK STENCIL INK.—Take of shellac two parts, borax one part, soft water ten parts, gum arabic one part, lampblack sufficient quantity, indigo sufficient quantity. Boil the shellac and the borax in the water until they are dissolved, add the gum arabic, and withdraw the mixture from the fire. When cold, add lampblack to bring it to a suitable color and consistence, and lastly, a very small quantity of finely-powdered indigo to give it a "jet" shade. Keep in glass or earthenware vessels.

---

PREPARATION OF WASHING BLUE.—Twenty lbs. white potato starch, twenty lbs. wheat starch, twenty lbs. Prussian blue, two lbs. indigo carmine, and two lbs. finely ground gum arabic are mixed in a trough, with the gradual addition of sufficient water to form a half-fluid homogeneous mass, which is poured out on a board with strips tacked to the edges. It is then allowed to dry in a heated room until it does not run together again when cut. It is next cut, by a suitable cutter, into little cubes, and allowed to dry perfectly. They are finished by being placed in a revolving drum, with a proper quantity of dry and finely pulverized Paris blue, until they have a handsome appearance. The cost is about 12 cents per lb.

---

GUMMED ADHESIVE PAPER.—Paper in sheets half of which are gummed on both sides, and the other half on one side, and divided into strips and squares of different sizes by perforations, like sheets of postage stamps, are very convenient in many ways—the doubly-gummed answering for fixing drawings in books, labels on glass, etc. It is stated that the mixture by which it is coated, is prepared by dissolving six parts of glue, previously soaked for a day in cold water, two parts of sugar, and three parts of gum arabic, in twenty-four parts of water, by the aid of heat.

---

TO PREVENT SPLITTING OF HANDLES.—All carpenters know how soon the butt ends of chisels split, when daily exposed to the blow of a mallet or hammer. A remedy suggested by a Brooklyn man consists simply in sawing or cutting off the round end of the handle so as to make it flat, and attaching by a few small nails on the top of it two round disks of sole leather, so that the end becomes similar to the heel of a boot. The two thicknesses of leather will prevent all further splitting, and if in the course of time they expand and overlap the wood of the handle, they are simply trimmed off all around.

---

VEGETABLE MARROW PRESERVE.—This makes a nice substitute for preserved ginger. Peel and cut the marrow in four, take out the seeds, then cut it into pieces about the size of a finger; sprinkle fine white sugar over it, and let it stand until it makes some liquid. Use equal weight of sugar to that of the cut marrow. Boil the liquid from the marrow along with the rest of the sugar and sliced lemons (one lemon to each pound of sugar,) until the syrup begins to thicken. Then put in the pieces of marrow and boil all for 20 to 30 minutes. When cool, add essence of ginger; quarter of an ounce to each pound of marrow is generally found the right quantity; but as tastes differ, it may be increased or lessened as desired.

---

BAG-MARKING INK.—A correspondent of the *English Mechanic* gives the following recipe for an ink, the permanency of which he says is perfect, even when bags filled with chemical manures have been in rain and sunshine over ten days: Boil 1 lb. of logwood chips in 1 gallon of water at boiling point ten minutes, then stir in the eighth of an ounce of bichromate of potash, and boil this ten minutes longer; then add, when cold, ½ lb. of common gum, previously dissolved, and stir well in. This will flow well from the pen, and will mark bags with either the stencil plate or block. The cost of the above ink is about 6d. (12 cents) per gallon.

---

TO REMOVE MILDEW.—We have several times been asked for a reliable recipe for the removal of mildew. We doubt whether there is any method that is infallible in all cases; but the following, which we find in an English journal, will often prove effectual: "Make a very weak solution of chloride of lime in water (about a heaped-up tea-spoonful to a quart of water,) strain it carefully, and dip the spot on the garment into it; and if the mildew does not disappear immediately, lay it in the sun for a few minutes, or dip it again into the lime-water. The work is effectually and speedily done, and the chloride of lime neither rots the cloth nor removes delicate colors, when sufficiently diluted, and the articles rinsed afterwards in clear water."

---

WATER-PROOFING WOVEN FABRICS.—The London *Chemist and Druggist* says that a good process for water-proofing cloths is to dip them in a solution of sulphate of alumina or alum cake, and afterwards in a warm solution of white Castile or curd soap. Ordinary soap may answer for dark cloths, but white soap should be used for white or light-colored goods. The cloths are then rinsed in clear cold water, and allowed to dry. The chemical reaction is simple enough: soaps are mainly composed of stearate of soda, which, on being rinsed with sulphate of alumina, forms sulphate of soda; that disappears in the washing, and an insoluble stearate of alumina is formed, which remains on the cloths and renders them water-proof.

---

MILK OF ROSES.—The following is a good recipe for this popular cosmetic:—

Almonds (blanched), ½ lb.;
Rose water, 1 qt.;
Alcohol, 95 per cent, 4 oz.;
Oil of rose, 1 dr.;
White wax,
Spermaceti,
Oil soap, of each, ½ oz.

Melt the soap in three ounces of rose water over a water bath, when melted add the wax and spermaceti, and mix well with occasional stirring; make an emulsion of milk with the almonds and rose water, and strain without pressure; place the saponaceous mixture in a mortar and with constant stirring gradually add the almond emulsion, and continue to stir until the whole is completely blended together. Next slowly add the alcohol (in which the oil of rose has been dissolved,) and stir vigorously for some time; put the whole in a bottle, let it stand twenty-four hours, then bottle for use, rejecting the sediment.

All the above precautions being taken, the milk of roses will keep a great length of time without any separation or precipitate.

---

TO COAT CASTINGS WITH COPPER.—The article should first be rendered free from rust by rubbing with an emery cloth, or by dipping into a pickle composed of sulphuric acid 2 oz., hydrochloric acid 1 oz., water 1 gallon. After the article has remained some time in this pickle it should be taken out, and the rust removed by a brush and some wet sand; if the oxide cannot be easily cleaned off, it must be returned to the pickle. As soon as the article is rendered bright, it is washed in a warm solution of soda or potash, for the purpose of removing all grease. Lastly, it is well rinsed in hot water, and immediately placed in a concentrated solution of sulphate of copper, to which a little sulphuric acid has been added. In a short time it will be coated with an even covering of metallic copper.

---

TO IMPROVE HONEY.—A Pittsburg correspondent of the *Scientific American* furnishes the following useful facts:

"On page 132 of your current volume, I noticed that one of your correspondents has great difficulty in preserving strained honey. Perhaps it would be of interest to him, as well as other readers of your valuable paper, to know that candied or crystallized honey can be permanently restored to transparency by the following method, which I have found successful: Take a flat-bottomed pan, as deep as the bottles containing the honey, and fill it with cold water; place the bottles in it so as not to touch each other; put it on a slow fire, and heat it up to 212° Fahr., and keep it at that heat until the honey is clear. Remove the pan from the fire, and you will have no further trouble with the honey."

---

TO FRESHEN FLOWERS.—A pinch of powdered sal-ammoniac in clear water in which cut flowers are put will keep them fresh for a long time.

